# Toll-Like Receptor-Dependent Immunomodulatory Activity of Pycnogenol^®^

**DOI:** 10.3390/nu11020214

**Published:** 2019-01-22

**Authors:** Annelies Verlaet, Nieke van der Bolt, Ben Meijer, Annelies Breynaert, Tania Naessens, Prokopis Konstanti, Hauke Smidt, Nina Hermans, Huub F.J. Savelkoul, Malgorzata Teodorowicz

**Affiliations:** 1 Department of Pharmaceutical Sciences, Laboratory of Nutrition and Functional Food Science, University of Antwerp, 2610 Wilrijk, Belgium; annelies.verlaet@uantwerpen.be (A.V.); Annelies.breynaert@uantwerpen.be (A.B.); Tania.naessens@UAntwerpen.be (T.N.); nina.hermans@uantwerpen.be (N.H.); 2 Department of Cell Biology and Immunology, Wageningen University & Research, 6708 WD Wageningen, The Netherlands; n.vanderbolt@sanquin.nl (N.v.d.B.); ben2.meijer@wur.nl (B.M.); huub.savelkoul@wur.nl (H.F.J.S.); 3 Laboratory of Microbiology, Wageningen University& Research, 6708 WE Wageningen, The Netherlands; prokopis.konstanti@wur.nl (P.K.); hauke.smidt@wur.nl (H.S.)

**Keywords:** Pycnogenol^®^, catechin, gastrointestinal metabolism, metabolites, Toll-like receptors, immunomodulation, partial agonist

## Abstract

Background: Pycnogenol^®^ (PYC), an extract of French maritime pine bark, is widely used as a dietary supplement. PYC has been shown to exert anti-inflammatory actions via inhibiting the Toll-like receptor 4 (TLR4) pathway. However, the role of the other receptors from the TLR family in the immunomodulatory activity of PYC has not been described so far. Aim: The aim of this study was to investigate whether PYC might exert its immunomodulatory properties through cell membrane TLRs (TLR1/2, TLR5, and TLR2/6) other than TLR4. Moreover, the effect of gastrointestinal metabolism on the immunomodulatory effects of PYC was investigated. Findings: We showed that intact non-metabolized PYC dose-dependently acts as an agonist of TLR1/2 and TLR2/6 and as a partial agonist of TLR5. PYC on its own does not agonize or antagonize TLR4. However, after the formation of complexes with lipopolysaccharides (LPS), it is a potent activator of TLR4 signaling. Gastrointestinal metabolism of PYC revealed the immunosuppressive potential of the retentate fraction against TLR1/2 and TLR2/6 when compared to the control fraction containing microbiota and enzymes only. The dialyzed fraction containing PYC metabolites revealed the capacity to induce anti-inflammatory IL-10 secretion. Finally, microbially metabolized PYC affected the colonic microbiota composition during in vitro gastrointestinal digestion. Conclusions: This study showed that gastrointestinal metabolism of PYC reveals its biological activity as a potential inhibitor of TLRs signaling. The results suggest that metabolized PYC acts as a partial agonist of TLR1/2 and TLR2/6 in the presence of the microbiota-derived TLR agonists (retentate fraction) and that it possesses anti-inflammatory potential reflected by the induction of IL-10 from THP-1 macrophages (dialysate fraction).

## 1. Introduction

The herbal extract Pycnogenol^®^ (PYC) is derived from the bark of French maritime pine (*Pinus pinaster* Aiton) [1]. PYC is standardized to contain 70 ± 5% (*w*/*w*) procyanidins, flavonoid oligomers, and polymers consisting of two to 12 units of catechin (CAT) and epicatechin. CAT, epicatechin, and taxifolin represent the flavonoids in PYC, of which CAT is the most common. Common dimers are procyanidin B1 (epicatechin-(4β→8)-catechin) and B3 (catechin-(4α→8)-catechin). Phenolic acids present in PYC are derivatives of benzoic acid (p-hydroxybenzoic acid, protocatechuic acid, gallic acid, vanillic acid) or cinnamic acid (caffeic acid, ferulic acid, p-cumaric acid) [2]. Known bioavailable compounds of PYC are CAT, caffeic acid, ferulic acid, taxifolin, and M1 (δ-(3,4-dihydroxy-phenyl)-γ-valerolactone) [3]. M1 is not a constituent of PYC, but is a metabolite of CAT produced by the gut microbiota upon ingestion [4].

PYC is widely used as a dietary supplement as it has been shown to exert multiple putative biological and pharmacological effects in various health conditions, next to its well-documented antioxidant and free radical scavenging properties [5,6,7,8,9,10]. PYC reduces the release of proinflammatory cytokines from macrophages and results in increased Foxp3 and IL-10 gene expression that is required for TReg development and functioning [7,11,12,13,14,15,16]. In addition, PYC reduces the expression of cell adhesion molecules (intercellular adhesion molecule (ICAM) and vascular cell adhesion molecule VCAM) [17], matrix metalloproteinase-9 (MMP-9) secretion [13,18,19], cyclo-oxygenase (COX) and lipoxygenase (LOX) expression [13,18,19,20,21,22], and levels of nitric oxide (NO) and inducible nitric oxide synthase (iNOS) expression [7,8,23,24]. Recent research highlighted that PYC exerts its immunomodulatory effects, at least in part, through the Toll-Like Receptor (TLR)4-NF-κB pathway [13,16,25,26,27]. Nevertheless, the mechanism by which PYC exerts its inhibitory effects via TLR4 and whether other TLR receptors and signaling mechanisms play a role remain to be elucidated.

Intestinal epithelial cells express TLRs recognizing microbiota and dietary compounds. These immune receptors are crucial to maintaining intestinal homeostasis and inducing the release of both pro- (tumor necrosis factor (TNF)-α, interleukin (IL)-1β, IL-6) and anti-inflammatory (IL-10) cytokines controlling inflammation [28]. Components present in the PYC extract are able to promote or compromise the local microenvironment in which mucosal immune responses are modified and the epithelial barrier is damaged or enforced.

The aim of this study was to elucidate whether PYC and its metabolite CAT exert immunomodulatory effects through membrane TLRs. In this study, we showed that non-metabolized PYC acts as an agonist of TLR1/2 and TLR2/6 and a partial agonist of TLR5, and exerts a dose-dependent induction of proinflammatory cytokine secretion (IL-1β, IL-8, and TNF). Microbial metabolism of this extract reflecting gastrointestinal exposure resulted in contact between PYC metabolites and TLR-expressing macrophages, potentially inducing immune activation. The digestion of PYC in the presence of enzymes and microbiota resulted in increased IL-10 production and alterations in microbiota indicative of immunomodulatory activity of the PYC hydrolysates.

## 2. Materials and Methods

### 2.1. Preparation of Samples and PYC-LPS Complexes

PYC was obtained from Horphag Research Ltd. (Geneva, Switzerland), and (+)-CAT hydrate (>98%) was purchased from Sigma Chemical Co. (St. Louis, MO, USA). For testing different solvents for PYC, the spray-dried PYC and CAT stocks were freshly dissolved in Phosphate Buffered Saline (PBS; Lonza, Verviers, Belgium), dimethyl sulfoxide (DMSO), or ethanol (96%) at the concentration of 50 mg/mL; vortexed for 1 min; and spun down for 1 min at 1000× *g*, room temperature (RT). The supernatant was sterilized by filtration using 0.22 µm Millex^®^-GV filters (Millipore Corporation, Bedford, MA, USA). Further dilutions of the samples were made in different types of the culture medium. PYC-lipopolysaccharide (LPS) complexes were made using a stock solution containing 400 µg/mL PYC, which was mixed with 50 pg/mL LPS from *Escherichia coli* 055:B5 (Sigma Aldrich, Zwijndrecht, The Netherlands). This stock dilution was further diluted in medium containing 50 pg/mL LPS and incubated for 1 h at RT to allow complex formation. Recombinant Factor C Endotoxin Detection Assay (cat. #609050, Hyglos GmbH, Bernried, Germany) was used to compare the LPS levels of the PYC-LPS solution and only LPS at the same concentration. The same assay was used for quantitative determination of LPS contamination in the PYC and CAT extracts. Recombinant Factor C Endotoxin Detection Assay is a homogenous enzymatic assay, using recombinant Factor C in combination with a fluorogenic substrate. Recombinant Factor C is the LPS receptor of the blood-clotting cascade in horseshoe crabs. The detection range of the assay is 0.005–50 EU/mL (equivalent of 0.5–1000 pg/mL). The assay was performed according to the manufacturer’s instructions. Results were obtained using SoftMax Pro software (Molecular Devices, LLC.; San Jose, CA, USA) and a newly calculated standard curves. Samples were spiked with 5 EU/mL LPS to validate if sample components interfered with the assay and heated to exclude false positive results by protease contamination of the samples. To re-calculate the LPS units from EU to pg/mL, we used the value 1 EU, which is approximately equivalent to 100 pg of LPS.

### 2.2. Gastrointestinal Dialysis Model

PYC was metabolized by a gastrointestinal dialysis model with colon phase (GIDM-colon) to mimic the in vivo situation after oral ingestion. The experimental set up was based on an in vitro continuous flow dialysis model, as described by Breynaert et al. [29]. Briefly, physiological conditions of the gastric phase were simulated for 1h at pH 2 with pepsin solution (37 °C, shaking conditions). The intestinal phase was simulated using Amicon stirred cells with a semi-permeable dialysis membrane (1000 Da cut-off) in an anaerobic glove-box (5% CO_2_, 5% H_2_ and 90% N_2_) at 37 °C. Dialysis mimics one-way GI absorption by passive diffusion from the lumen to mucosa. The small intestine was mimicked for 2.5 h at 37 °C and pH 7.5 with pancreatic enzymes and bile solution. Afterwards, the colonic phase with retentate samples from the small intestine was simulated at pH 5.8 and by the addition of microbial faecal culture obtained from pooled faeces from healthy adult donors. Both retentate and dialysate samples were taken at specific time points and freeze-dried prior to analysis. GIDM digestion was performed in duplicate for PYC (starting dose of 0.024 g up to a high dose of 0.2 g), in addition to a blank without PYC and a blank without faecal suspension.

### 2.3. High-Performance Liquid Chromatography (HPLC) Analysis

As the high concentration of PYC used in this study did not dissolve completely in PBS, HPLC analysis for the identification of CAT, caffeic acid, taxifolin, and ferulic acid, as well as the detection of the overall percentage of procyanidins by spectrophotometry, were performed according to the United States Pharmacopeial (USP 38) methods for Maritime Pine Extract. For fingerprint HPLC, PYC was, however, dissolved in 20% methanol (instead of 100% as described in the United States pharmacopeia (USP)), optimizing resulting chromographic peaks.

### 2.4. Culture of Human Embryonic Kidney (HEK 293) Cells

Human embryonic kidney (HEK 293) cells stably transfected with TLR1/TLR2, TLR2/TLR6, TLR4, TLR5, and the empty pNIFTY plasmids were obtained from CAYLA-InvivoGen (Toulouse, France). TLR activation upon ligand stimulation was determined by assessing nuclear-factor-kappaB (NF-κB) activation. The latter was achieved by stably transfecting the HEK 293 cells with pNIFTY, a family of NF-κB inducible reporter plasmids expressing the secreted alkaline phosphatase reporter gene. The selective antibiotics Zeocin^TM^ and Blasticidin (Invitrogen, Auckland, New Zealand) were used to generate stable clones. HEK 293 cells were cultured in Dulbecco’s Modified Eagle’s Medium (DMEM; ThermoFisher, Waltham, USA), supplemented with 1% Penicillin/Streptomycin (P/S), 10% Foetal Bovine Serum (FBS), and 2 mM l-Glutamine from Gibco (Invitrogen, Auckland, New Zealand), and incubated at 37 °C in 5% CO_2_ in a humidified incubator.

For the activation of TLR receptors, human embryonic kidney (HEK 293) cells were seeded (6 × 10^4^ cells/well) in Costar^®^ 3610 white clear flat-bottom 96 well plates (Corning Inc., Corning, NY, USA) and cultured for 24 h. Cells were stimulated with different concentrations of PYC (400, 100, 25, 6.25 µg/mL) and CAT (250, 50, 5, 0.5 µg/mL) or metabolized samples taken at specific time points (4 h, 10 h, 27 h and 36 h) and diluted in the culture medium. Their known ligands were used as positive controls: Triacylated lipopeptide (PAM3CSK4, #tlrl-pms, InvivoGen, San Diego, CA, USA), PAM2 (PAM2CSK4, #tlrl-pms, InvivoGen, San Diego, CA, USA), LPS from *Escherichia coli* 055:B5 (#L2880, Sigma Aldrich, Zwijndrecht, The Netherlands), and flagellin (#vac-fla, InvivoGen, San Diego, CA, USA) for TLR1/2, TLR2/6, TLR4, and TLR5, respectively. After 18 h of incubation, the luciferase activity in HEK 293 cells was measured and related to the cells grown in the medium as a control. Bright-Glo Luciferase Assay System (#E2610, Promega, Madison, WI, USA) was added to medium (1:1) and vortexed for 5 min at 250 rpm before luminescence was read from the top with a 750 ms integration time using a Microplate Reader (Molecular Devices, Sunnyvale, CA, USA).

### 2.5. Culture of Human Acute Monocytic Leukaemia Cell Line (THP-1)

The human acute monocytic leukaemia cell line, THP-1 (American Type Culture Collection, Manassas, VA, USA), was maintained in RPMI 1640 medium with L-Glutamine and 25 mM Hepes (Lonza, Verviers, Belgium), supplemented with 10% FBS and 1% P/S. Cells were sub-cultured twice, and medium was changed before every experiment. Cells were incubated at 37 °C in a humidified atmosphere with 5% CO_2_ and were used below passage number 25. THP-1 cells were seeded at 1 × 10^5^ cells/well in Costar^®^ 3596 flat bottom 96-well plates (Corning Inc., Corning, NY, USA). Macrophage phenotype was induced by stimulating THP-1 cells with 10 ng/mL PMA (Sigma Aldrich, Zwijndrecht, The Netherlands) for 48 h. Differentiated cells were washed twice in culture medium and incubated for another 48 h before stimulation. Afterwards, the cells were stimulated with different concentrations of PYC (400, 100, 25, 6.25 µg/mL) and CAT (250, 50, 5, 0.5 µg/mL) or metabolized samples taken at specific time points (4 h, 10 h, 27 h, and 36 h) and diluted in the culture medium. These optimized doses were obtained from pilot experiments in which more doses between 100 and 400 mg/mL were tested. The experiment was performed with and without co-stimulation with PAM3 at a concentration of 0.1 µg/mL. After 18 h of incubation, the supernatant was collected and human cytokine concentrations (IL-6, IL-8, IL-1β, TNF-α, and IL-10) were determined using the cytometric bead array (CBA) kit (Human Inflammatory Cytokine Kit, cat. #551811, BD Bioscience, Franklin Lakes, NJ, USA), according to the manufacturer’s instructions. The samples were analyzed by flow cytometry (BD FACS Canto II, BD Bioscience). The results were normalized to cytokine levels of unstimulated macrophages cultured in the medium.

### 2.6. Cytotoxicity Assay

Cytotoxicity of PYC and CAT solutions was determined using the CellTiter 96 Aqueous One Solution Cell Proliferation assay (cat. #G3580, Promega, Madison, WI, USA) and both the HEK 293 cell line and THP-1 macrophages. Cells were plated at a density of 1 × 10^5^ cells/well for THP-1 and 6 × 10^4^ cells/well for HEK 293 cells in flat bottom 96-well plates (Corning Inc., Corning, NY, USA) and incubated with PYC and CAT extracts at different concentrations for 18h. Afterwards, 15 µL of CellTiter96^®^ Aqueous One Solution was added and plates were incubated further for 1h at 37 °C in a humidified atmosphere with 5% CO_2_. After incubation, absorbance was recorded at 485 nm using the FilterMax F5 Multi-Mode Microplate Reader (Molecular Devices, Sunnyvale, CA, USA). Viability of cells stimulated with sample conditions was normalized in relation to the negative control (cells cultured in pure medium). All experiments were performed in triplicate.

### 2.7. Expression of TLRs on THP-1 Macrophages

THP-1 macrophages were incubated for 18 h, with dialysates collected after 4 and 10 h of metabolism without or with triacylated lipopeptide (PAM3CSK4, #tlrl-pms, InvivoGen, San Diego, CA, USA) for co-stimulation and as described before [30]. After 18 h of incubation, the cells were harvested, and the expression of receptors was analyzed (CD83, TLR1, TLR5, and TLR6) using FACS CantoII (BD Pharmingen, Franklin Lakes, NJ, USA). The following antibodies were used: anti-human CD83 (#305306 BioLegend, San Diego, CA, USA), TLR1 (#565792, BD Pharmingen, Franklin Lakes, NJ, USA), TLR5 (#564344 BD Pharmingen), and TLR6 (#566339, BD Pharmingen, Franklin Lakes, NJ, USA).

### 2.8. Microbiota Analyses

Bacterial DNA was isolated from the retentate samples, which were collected from the colonic phase at time 4 and 10 h. The samples were added to a bead-beating tube with 300 µL of Stool Transport and Recovery (STAR) buffer, 0.25 g of sterilized zirconia beads (0.1 mm), and three glass beads (2.5 mm). Samples were homogenized by three lots of bead-beating (60 s × 5.5 ms) and incubated for 15 min at 95 °C. Samples were then centrifuged for 5 min at 4 °C (14,000× *g*), and supernatant was collected and transferred to sterile tubes. Pellets were re-processed, this time adding 200 µL of STAR buffer (Roche), and supernatants from both steps were pooled. DNA purification was performed with a customized kit (XAS 1220; Promega) using 250 µl of the final supernatant pool. DNA was eluted in 50 µL of DNAse- RNAse-free water, and its concentration was measured using the DS-11 FX+ Spectrophotometer/Fluorometer (DeNovix Inc., Wilmington, DC, USA) [31]. For the amplification of the V5-V6 region of the bacterial 16S rRNA gene, DNA concentration was adjusted to 20 ng/µL. PCR conditions were set as described previously [32]. Amplicons were pooled, and sequencing was performed by Illumina HiSeq sequencing at GATC (Konstanz, Germany).

Real-time PCR amplification and detection were performed on a CFX384™ real-time PCR detection system (Bio-Rad, Hercules, CA, USA). Broad range primers were used, targeting the bacterial 16S rRNA gene [33]. The reaction mixture was composed of 5 μL iQ™ SYBR^®^ Green Supermix, 0.2 μL forward and reverse primers (10 nmol), 1.6 μL nuclease-free water, and 3 μL of DNA template (1 ng/μL). The program for amplification was initial denaturation at 94 °C for 5 min, followed by 40 cycles of denaturation at 94 °C for 20 s, annealing at 52 °C for 30 s, and elongation at 72 °C for 30 s. Standard curves contained 10^1^–10^9^ 16S rRNA copies/μL and were included in triplicate.

### 2.9. Data Normalization and Statistical Analysis

Statistical analysis on cell cultures in vitro was determined in software by one-way ANOVA with a Tukey post hoc comparison test (GraphPad Prism). All data are expressed as the mean ± standard deviation (SD). *p*-values < 0.05 are considered statistically significant. Significant differences are indicated by asterisks: * *p* < 0.05; ** *p* < 0.01; *** *p* < 0.001.

Sequencing data filtering and taxonomy assignment were performed using the NG-Tax pipeline using the default settings [32]. Taxonomy was assigned to the identified Operational Taxonomic Units (OTU) using the Silva_111_SSU database [34]. The total number of obtained reads was 266972, while 33912 was the minimum number of reads obtained for a sample. The microbiota data were analyzed at the genus level and transformed into relative abundances prior to downstream analysis. In total, 32 bacterial genera were identified from the four samples.

### 2.10. Data Availability

The 16S rRNA gene sequencing dataset is publicly available at the European Nucleotide Archive (ENA) database with accession code PRJEB29793.

## 3. Results

### 3.1. Solubility of the Specific Monomers in the PYC Solution

PYC was dissolved at the concentration of 50 mg/mL in three different solvents: ethanol (96%), DMSO, and PBS. The cytotoxicity of the PYC solutions was then determined using the HEK 293 cell line, as well as THP-1 macrophages. DMSO, even at low concentrations, was found to significantly decrease the viability of both cell lines and therefore PYC solution in DMSO was not used for further experiments. PBS and ethanol solutions of PYC were tested on HEK cells expressing TLR4, TLR1/2, TLR2/6, and TLR5 receptors. It was found that the ethanol solution impaired the valid TLR modulation of TLR1/2 and TLR5 receptors (Figure S1) and therefore PBS was chosen as the most optimal solvent. Moreover, the use of an aqueous solvent like PBS mimics the in vivo situation after the oral administration of PYC.

**Figure 1:**
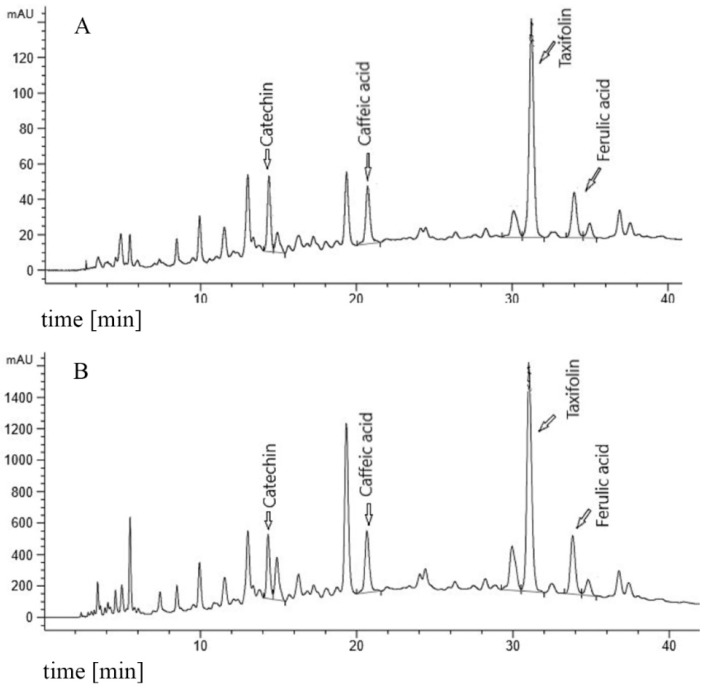
Fingerprint chromatogram. (**A**) 20 mg/mL PYC (Pycnogenol^®^) reference in 10% MeOH (methanol). (**B**) 50 mg/mL PYC in PBS (Phosphate Buffered Saline).

In order to ensure that the composition of the PYC samples dissolved in PBS was sufficiently similar to the composition of a sample completely dissolved in methanol (MeOH), the solubility of the specific monomers in the PYC solutions was analyzed by HPLC. The analytical outcomes showed that the concentrations (in % of analysis of the MeOH solution) of the four major PYC monomeric polyphenols (CAT, caffeic acid, taxifolin and ferulic acid), as indicated in the HPLC fingerprint (Figure 1), do not significantly differ in the PBS solutions up to a concentration of 50 mg/mL, compared to the analysis in MeOH (=100%). Even for the highly concentrated stock solution, the solubility was found to be 80–92.2%, which did not significantly differ from the MeOH solution of PYC (Table 1). In addition, spectrophotometric analysis revealed that the dissolved extract contained 60.2% procyanidins as opposed to 78.3 ± 3% in the MeOH solution of PYC, also confirming a solubility of about 80% for procyanidins.

### 3.2. PYC and CAT are LPS Free and not Cytotoxic to HEK and THP-1 Cells

**Figure 2:**
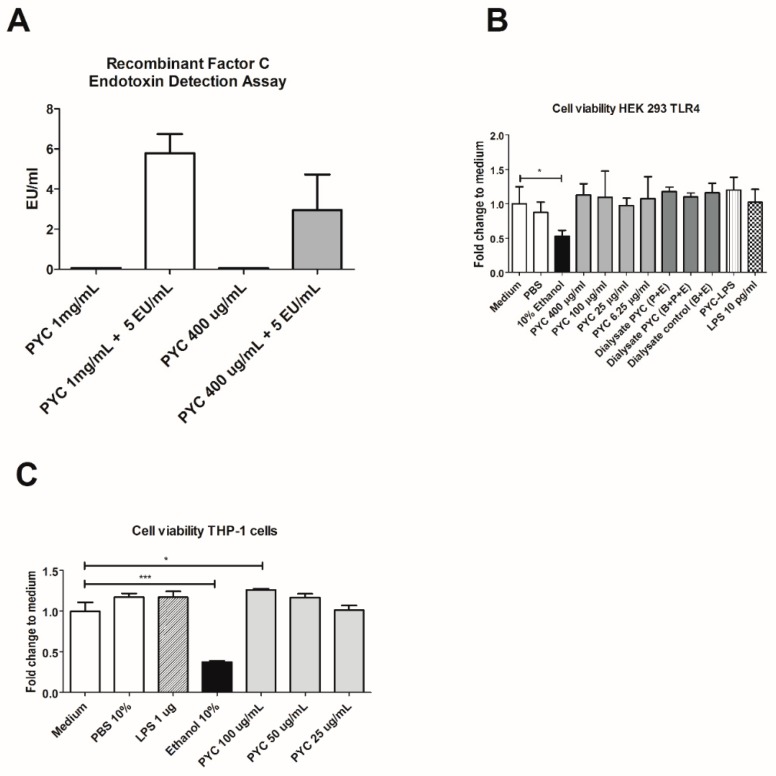
PYC extract contains non-detectable amounts of LPS and does not influence the viability of Human Embryonic Kidney (HEK)-Blue 293 and Human Acute Monocytic Leukaemia (THP-1) cell lines. (**A**) Lipopolysaccharide (LPS) concentration in Pycnogenol^®^ (PYC) extract without and after spiking with 5 EU/mL of LPS detected with EndoZyme recombinant factor C assay (*n* = 4 as technical replicates). Spiking recovery between 50% and 200% excludes interference of sample components with the assay. (**B**) Viability of HEK-293 cells expressed as fold change of absorbance (490 nm) compared to unstimulated cells cultured in medium as control (*n* = 4 as technical replicates). (**C**) THP-1 macrophages cultured for 24 h in the presence of PYC. Results are expressed as fold change of absorbance (490 nm) comparedto unstimulated cells cultured in medium as control (*n* = 4 as technical replicates). Ethanol was used as the positive control that affects cell viability. All data are expressed as the mean ± standard deviation (SD). *p*-values < 0.05 are considered statistically significant, as analyzed with one-way ANOVA with Tukey post hoc comparison test (GraphPad Prism). Significant differences are indicated by asterisks: * *p* < 0.05; *** *p* < 0.001.

The putative endotoxin (LPS) contamination of the PYC preparation was below the detection limit of 0.05 EU/mL, even at a high concentration of PYC (1 mg/mL) (Figure 2A), showing that the samples were essentially not contaminated with LPS. Cytotoxicity of all tested preparations (LPS, PYC, PYC-LPS and hydrolysates of PYC) at the concentrations corresponding with further experiments was examined for both the HEK and THP-1 cell line. None of the above-mentioned compounds were found to significantly decrease the viability of these two cell lines (Figure 2B,C).

### 3.3. PYC Acts as An Agonist of TLR1/2 and TLR2/6 and a Partial Agonist of TLR5

**Figure 3:**
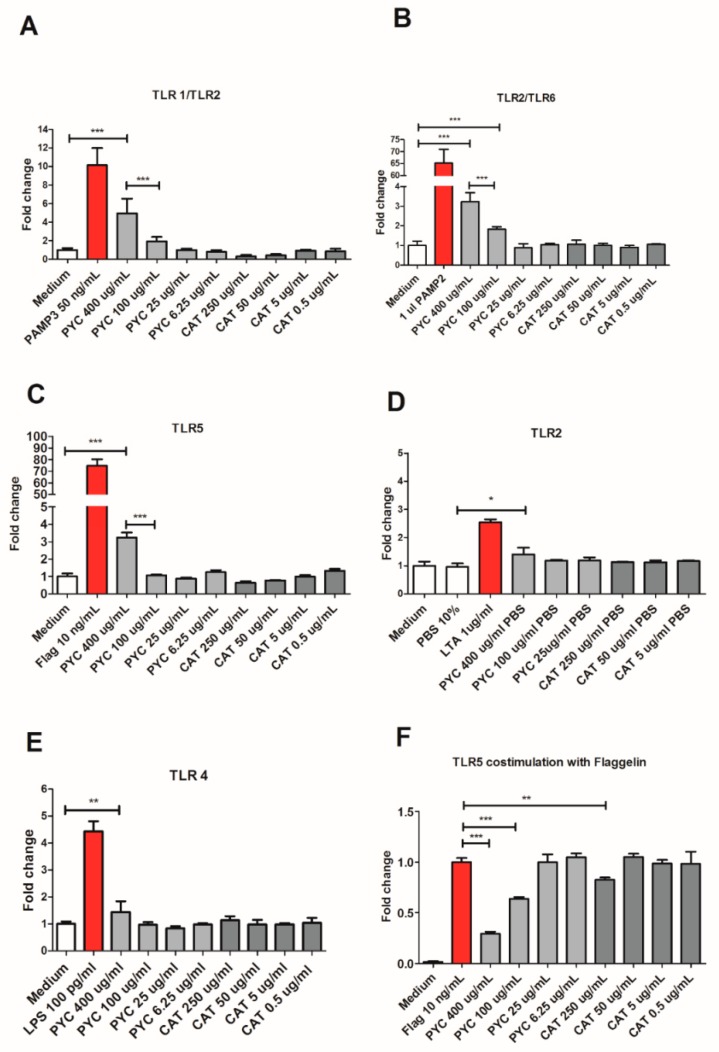
PYC extract acts as an agonist of Toll-Like Receptor (TLR)1/2 and TLR2/6 and as a partial agonist of TLR5. HEK 293 cells expressing the receptors (**A**) TLR1/2, (**B**) TLR2/6, (**C**) TLR5, (**D**) TLR2, and (**E**) TLR4 were incubated for 24 h with different concentrations of PYC and catechin (CAT) or their known ligands as positive controls (PAM3, PAM2, flagellin, lipoteichoic acid (LTA), and LPS, resp.). All corresponding positive ligands induced TLR activation, confirming functionality of the assays. (**F**) HEK 293 cells expressing TLR5 were incubated for 24 h with different concentrations of PYC and CAT in combination with its known ligand as positive control (flagellin). Results are expressed as fold change of fluorescence intensity to unstimulated cells cultured in medium as control (*n* = 4 as technical replicates) All data are expressed as the mean ± SD. *p*-values < 0.05 are considered statistically significant, as analyzed with one-way ANOVA with Tukey post hoc comparison test (GraphPad Prism). Significant differences are indicated by asterisks: * *p* < 0.05; ** *p* < 0.01; *** *p* < 0.001.

The agonistic effect of PYC and CAT was measured using stably transfected HEK 293 cell lines expressing the following TLR receptors: TLR2, TLR1/2, TLR2/6, TLR4, and TLR5. Responses of these cell lines were determined by quantifying luminescence upon selective ligand binding, induced by the pNIFTY reporter gene via the NF-κB pathway (Figure 3). The stimulation of cells with PYC extract showed a dose-dependent increase of luminescence of the cell lines expressing the heterodimeric receptors TLR1/TLR2 and TLR2/TLR6 (Figure 3A,B). The highest concentration of PYC (400 µg/mL) induced 4.9- and 3.2-fold activation for TLR1/TLR2 and TLR2/TLR6, respectively, when compared to the negative control being culture medium (Figure 3A,B). The highest concentration of PYC (400 µg/mL) induced the activation of TLR5, which was 3.2-fold higher than for the medium control; however, no dose dependency was observed (Figure 3C). Little activation of TLR2 (Figure 3D) and TLR4 (Figure 3E) was observed (around 1.4 higher than that observed for medium control) upon the incubation of HEK 293 cells with 400 µg/mL PYC. CAT, the main monomer of PYC, showed no agonistic effects on any of the membrane TLRs investigated, suggesting that CAT is not the constituent of PYC responsible for the observed activation of TLR1/TLR2 and TLR2/TLR6 (Figure 3A–D). Nevertheless, CAT dose-dependently reduced the activity of TLR5 (0.6- and 0.7-fold) and TLR1/TLR2 (0.3- and 0.4-fold) below the levels of the negative control being a medium. Moreover, PYC but not CAT was shown, in a dose-dependent manner, to induce proinflammatory cytokine (IL-8, TNF-α and IL1-β) secretion from THP-1 macrophages, confirming an agonistic effect on TLR signaling (Figure S2). To investigate whether PYC and CAT function as antagonists or partial agonists of studied TLRs, co-stimulation of each receptor with PYC/CAT and its positive control was performed. PYC dose-dependently inhibited the activation of TLR5 when co-stimulated with flagellin, reducing the level of activation to 0.29- and 0.63-fold in the studied concentrations of 400 and 100 µg/mL, respectively (Figure 3F), while CAT reduced the activation of TLR5 only at the highest concentration (250 µg/mL). Co-stimulation of the other receptors did not affect the level of receptors’ activation. Therefore, these data suggest that PYC extract acts as an agonist of TLR1/2 and TLR2/6 and as a partial agonist of TLR5.

**Figure 4:**
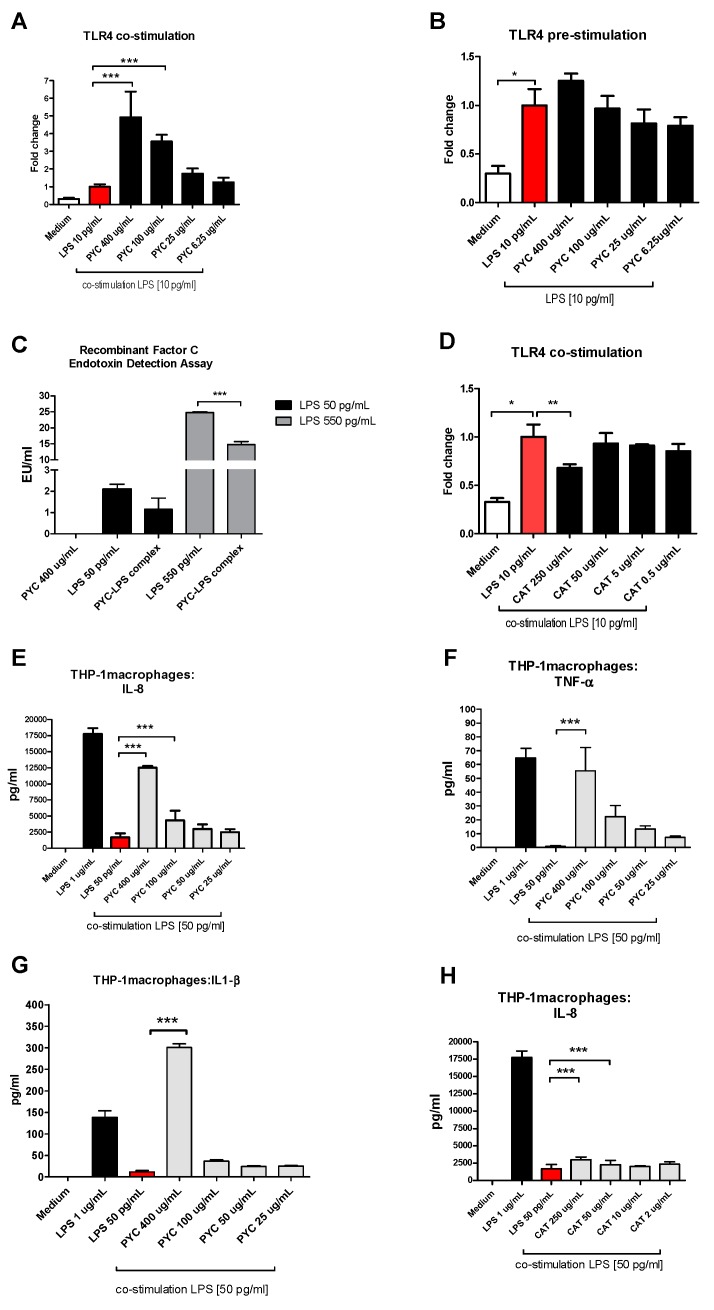
PYC and LPS form complexes, which boost activation of TLR4. (**A**) PYC was pre-incubated with LPS (10 ng/mL) 30 min before addition to the TLR4-expressing HEK cells, followed by 24 h incubation. (**B**) TLR4-expressing HEK cells were pre-incubated with PYC, followed by washing and incubation with LPS (10 ng/mL) for 24 h. (**C**) LPS concentration in pure PYC sample (400 µg/mL) and after 30 min incubation with LPS standard (50 and 550 pg/mL) expressed in LPS units EU/mL. (**D**) CAT was pre-incubated with LPS (10ng/mL) for 30 min before addition to the TLR4-expressing HEK cells for 24 h. Results of A, B, and D are expressed as fold change of fluorescence intensity to the cells stimulated with 10 ng/mL LPS. Concentration of (**E**) interleukin (IL)-8, (**F**) tumor necrosis factor (TNF)-α, and (**G**) IL-1β in supernatants of THP-1 macrophages after incubation with LPS (1 and 50 pg/mL) and with PYC-LPS complexes formed during 30 min pre-incubation of LPS (50 pg/mL) with different concentrations of PYC. ((**H**) Concentration of IL-8 in supernatants of THP-1 macrophages after incubation with LPS (1 and 50 pg/mL) and with CAT-LPS complexes formed during 30 min pre-incubation of LPS (50 pg/mL) with different concentrations of CAT. All data (*n* = 4 technical replicates from two independent experiments) are expressed as the mean ± SD. *p*-values < 0.05 are considered statistically significant, as analyzed with one-way ANOVA with Tukey post hoc comparison test (GraphPad Prism). Significant differences are indicated by asterisks: * *p* < 0.05; ** *p* < 0.01; *** *p* < 0.001.

Interestingly, a clear stimulating dose-response curve was observed for PYC on TLR4 activity when TLR4-expressing HEK cells were stimulated with a mixture of PYC and LPS (10 pg/mL). It was shown that 400, 100, and 25 µg/mL PYC in combination with LPS induced a 4.9-, 3.5-, and 1.7-fold increase in TLR4 activation, respectively, when compared to pure LPS at the same concentration (10 pg/mL) (Figure 4A). As shown above, PYC on its own, at the concentration of 400 µg/mL, significantly induced TLR4 activation, albeit at a low level (Figure 3E). Also, pre-incubation of cells with similar doses of PYC followed by stimulation with LPS did not influence the level of TLR4 activation when compared to the LPS control (Figure 4B). The observation of the increased activation of TLR4 by co-stimulation with the PYC-LPS mixture may be explained by the formation of complexes between PYC and LPS, beneficially affecting the activation of TLR4. This has been confirmed by a decreased concentration of LPS measured in the PYC-LPS preparation after 30 min of pre-incubation (Figure 4C). Co-stimulation of TLR4^+^ HEK cells with CAT and LPS did not show any boosting effect on TLR4 activation. CAT at a concentration of 250 μg/mL showed inhibiting activity against TLR4 activation, which was 30% lower than that of the LPS control (Figure 4D). The enhanced activation of TLR4 in HEK 293 cells by co-stimulation of cells with PYC and LPS was confirmed by the boosted cytokine secretion by THP-1 macrophages stimulated with PYC-LPS complexes (Figure 4E–G). Co-stimulation of cells with LPS and different doses of PYC resulted in higher levels of IL-1β, IL-8, and TNF-α when compared to cells stimulated with only LPS at the same concentration. When co-stimulated with LPS, 400 and 100 µg/mL PYC caused a 26- and 3-fold increase in the concentration of IL-1β, a 7- and 2.5-fold increase in IL-8 concentration, and a 75- and 21-fold increase of the levels of TNF-α, respectively. No effect of co-stimulation of CAT with LPS on the cytokine levels was observed (Figure 4H).

### 3.4. Gastrointestinal Hydrolysate of PYC Modulates Activation of TLRs

The PYC extract was subjected to gastrointestinal metabolism with and without the presence of human faecal suspension in the medium. Retentate and dialysate fractions from different time points (4 h, 10 h, 27 h, and 36 h) were tested for their potential to activate TLRs using both the HEK 293 cell model and cultured THP-1 macrophages.

**Figure 5:**
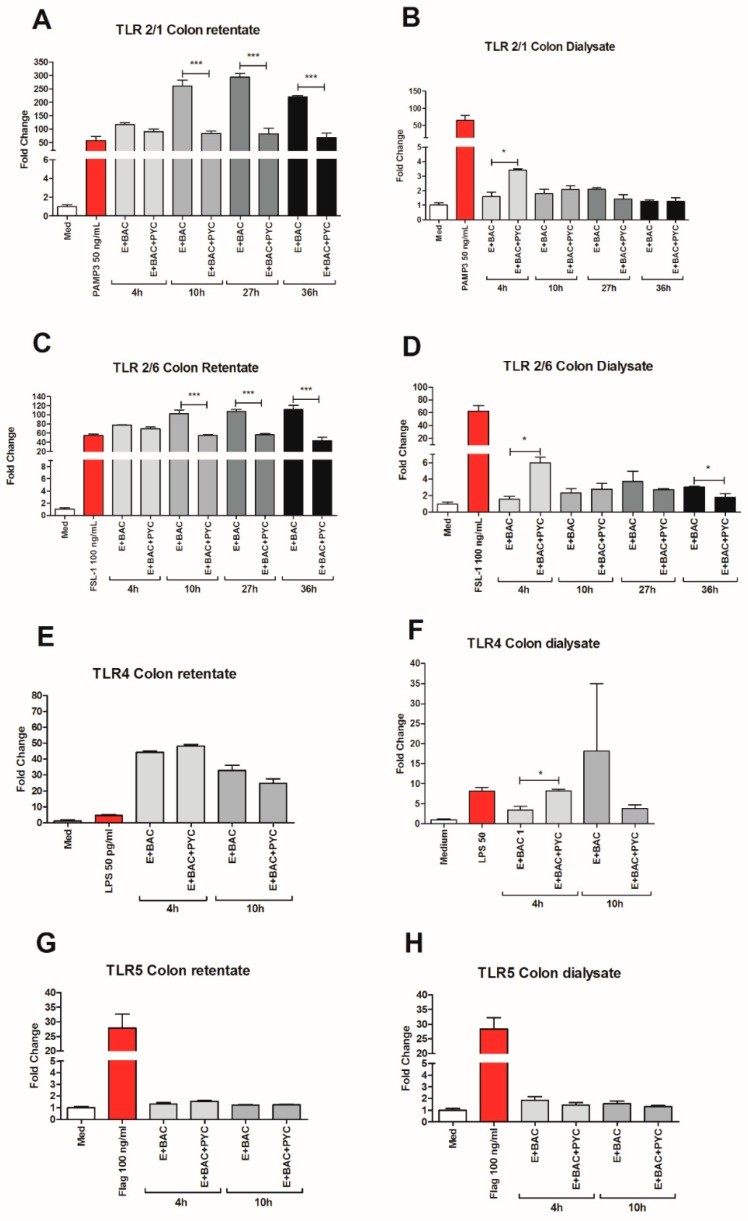
Gastrointestinal metabolism modulates the interaction of PYC with TLRs. PYC was subjected to gastrointestinal metabolism in the presence of human faecal suspension. In the intestinal phase, a semi-permeable dialysis membrane mimics one-way absorption. Both retentate and dialysate samples were taken at specific time points (4 h, 10 h, 27 h, and 36 h) and used for stimulation of the HEK 293 cells expressing (**A**,**B**) TLR1/2, (**C**,**D**) TLR2/6, (**E**,**F**) TLR4, and (**G**,**H**) TLR5, which were also stimulated with their known ligands as positive controls (PAM3, FSL-1, LPS, and flagellin, resp.). After 24 h of incubation, the luciferase activity in HEK 293 cells was measured and expressed as fold change of fluorescence intensity to unstimulated cells cultured in medium as control. PYC samples were metabolized in the presence of E: enzymes; BAC: intestinal bacteria (human faecal suspension). All data are expressed as the mean ± SD of *n* = 4 technical replicates from two independent experiments. *p*-values < 0.05 are considered statistically significant, as analyzed with one-way ANOVA with Tukey post hoc comparison test (GraphPad Prism). Significant differences are indicated by asterisks: * *p* < 0.05; *** *p* < 0.001.

Comparing the control retentate samples containing enzymes and bacteria with the test retentate samples containing PYC, enzymes, and bacteria, collected at time points 10 h, 27 h, and 36 h, showed a significant suppressive effect upon the stimulation of HEK 293 cells expressing TLR1/2 and 2/6 (Figure 5A,C). The control samples only stimulated TLR1/2 on average four times more than 50 ng/mL PAM3 positive control for time points 10 h, 27 h, and 36 h. This level of stimulation was reduced by the presence of PYC metabolites to a 1.4 higher stimulation as compared to the PAM3 positive control. The same significant reduction of receptor activation was observed upon the stimulation of HEK 293 cells expressing TLR2/6. No clear effect of the retentate containing PYC metabolites on the stimulation of HEK 293 cells expressing TLR4 and TLR5 was observed (Figure 5E,G).

When comparing the control dialysate fractions containing enzymes and bacteria only with the fractions enriched with PYC, significantly higher stimulation was observed by the fraction containing PYC metabolites collected at time point 4 h for the HEK 293 cells expressing TLR1/2, TLR2/6, and TLR4 (Figure 5B,D,F). These differences declined with time of metabolism, even showing an inverted tendency for TLR2/6, where the level of the activation of the receptor was significantly reduced by fractions enriched with PYC when compared to the control at 27 h. A similar (but not significant) tendency was also observed for TLR1/2 and TLR4. No effect of the dialysates containing PYC metabolites on the stimulation of HEK 293 cells expressing TLR5 was observed (Figure 5H).

### 3.5. Gastrointestinal Metabolites of PYC Induce IL-10 Production by THP-1 Macrophages

**Figure 6:**
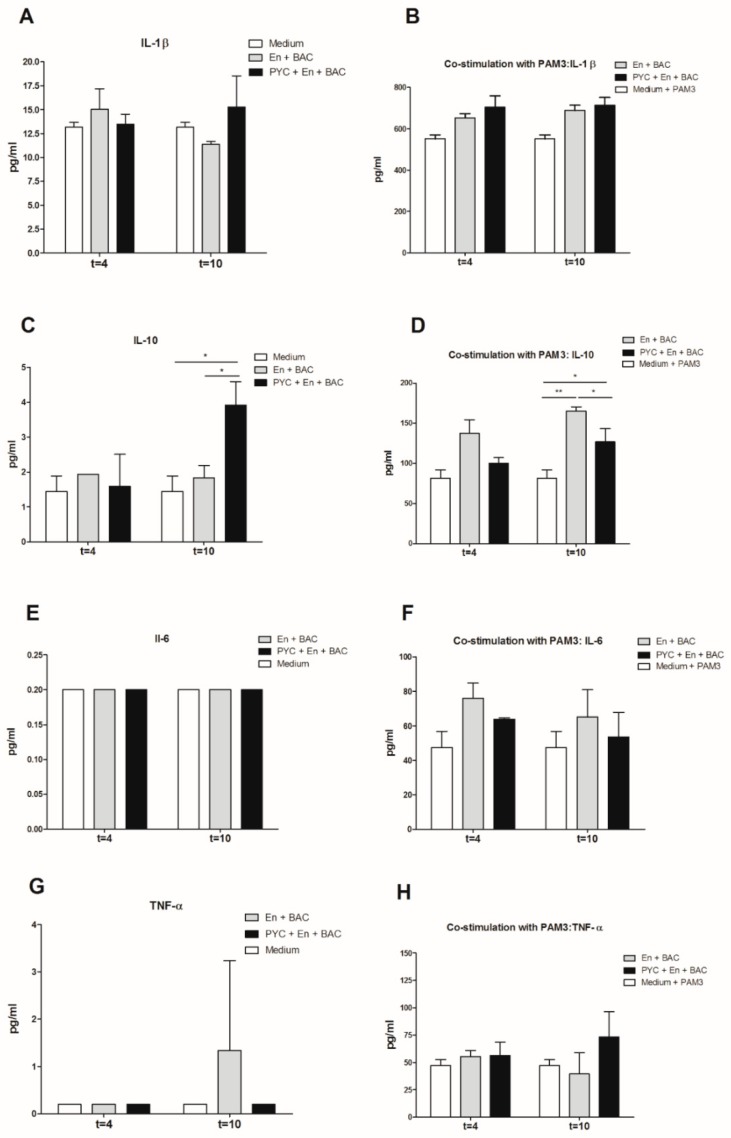
Proinflammatory activity of PYC is abolished by metabolism in the presence of human faecal suspension. THP-1 macrophages were incubated 18h with dialysates collected after 4 h and 10 h of metabolism with (**A**,**C**,**E**,**G**)) and without (**B**,**D**,**F**,**H**)) co-stimulation with the known ligand PAM3 at a concentration of 0.1 µg/mL. After 24 h of incubation the concentration of (**A**,**B**) IL-1β, (**C**,**D**) IL-10, (**E**,**F**) IL-6 (below detection limit on figure (**E**)), and (**G**,**H**) TNF-α in the supernatants was determined. PYC samples were metabolized in the presence of E: enzymes; BAC: intestinal bacteria (human faecal suspension). All data are expressed as the mean ± SD of *n* = 4 independent replicates. *p*-values < 0.05 are considered statistically significant, as analyzed with one-way ANOVA with Tukey post hoc comparison test (GraphPad Prism). Significant differences are indicated by asterisks: * *p* < 0.05; ** *p* < 0.01.

The dialyzed fractions containing PYC metabolites collected at time point 4 h and 10 h were incubated with THP-1 macrophages to study their immunogenicity by measuring the cytokine profile (Figure 6). The levels of TNF-α, IL-1β, IL-6, and IL-10 in the supernatants were evaluated after 18 h of incubation with or without PAM3 as a co-stimulant. Neither the control dialysate fraction containing enzymes and bacteria nor the dialysate fraction also containing PYC metabolites induced the secretion of any of the proinflammatory cytokines (IL-1β, IL-6, TNF-α, Figure 6A,E,G), while the dialysate fraction containing PYC metabolites significantly induced the production of IL-10 compared to the cells incubated with control dialysate fractions which did not contain PYC metabolites (Figure 6C). The significant induction of IL-10 was also observed in the presence of PAM3 for both the control dialysate containing enzymes and bacteria and the dialysate sample containing PYC metabolites. However, the samples containing PYC showed a significantly lower capacity to induce IL-10 when compared to the control. Nevertheless, both samples were shown to significantly shift the balance of cytokines produced by PAM3-stimulated macrophages towards regulatory IL-10 secretion (Figure 6D).

### 3.6. Gastrointestinal Metabolites of PYC Up-Regulate the Expression of CD83, TLR1, and TLR5

**Figure 7:**
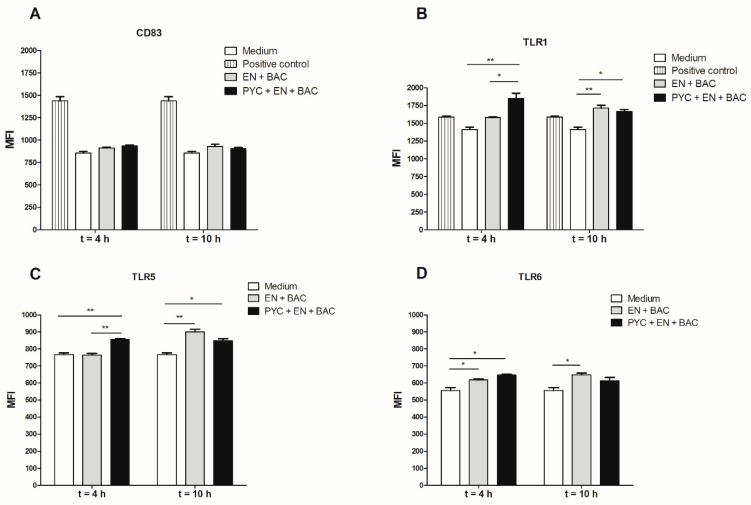
Metabolized PYC up-regulates the expression of cluster of differentiation (CD) 83, TLR1, TLR5, and TLR6. THP-1 macrophages were incubated for 18h with dialysates collected after 4 h and 10 h of metabolism or PAM3 as positive control. After 18h of incubation, the cells were harvested and the expression of (**A**) CD83, (**B**) TLR1, (**C**) TLR5, and (**D**) TLR6 was determined and expressed as differences in mean fluorescence intensity (MFI). PYC samples were metabolized in the presence of E: enzymes; BAC: intestinal bacteria (human faecal suspension). All data are expressed as the mean ± SD of *n* = 4 independent replicates. *p*-values < 0.05 are considered statistically significant, as analyzed with one-way ANOVA with Tukey post hoc comparison test (GraphPad Prism). Significant differences are indicated by asterisks: * *p* < 0.05; ** *p* < 0.01.

THP-1 macrophages were incubated for 18 h with dialysates of metabolized PYC samples, and the level of expression of the following receptors was evaluated: CD83, TLR1, TLR5, and TLR6 (Figure 7). A significantly enhanced TLR1 and TLR5 expression was observed upon incubation of the THP-1 macrophages with a dialysate fraction containing PYC metabolites when compared to the control dialysate fraction containing only enzymes and bacteria (Figure 7B,C). This effect was only visible for time point 4 h of gastrointestinal metabolism and was abolished after 10 h of metabolism. The expression of the co-stimulatory molecule CD83 on the surface of THP-1 macrophages was not affected by the dialysate fraction containing PYC metabolites or by the control fraction.

### 3.7. Gastrointestinal Metabolites of PYC Modulate Microbiota Composition

**Figure 8:**
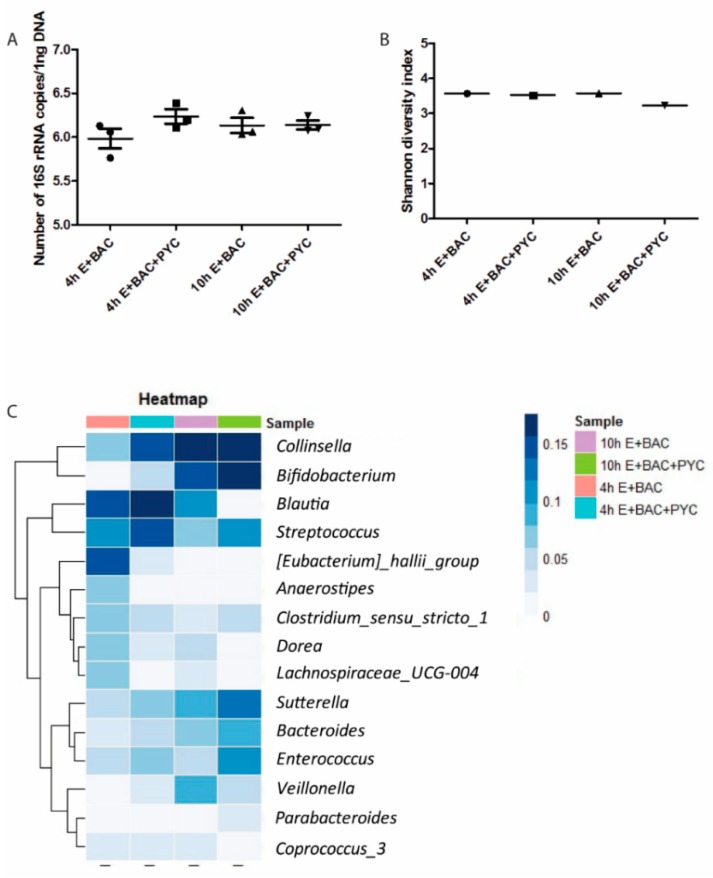
PYC metabolites modulate microbiota composition. (**A**) Absolute abundance of total bacteria present in the four samples, determined by quantitative polymerase chain reaction (qPCR), with total counts log 10 transformed. (**B**) Alpha diversity of the four samples calculated using the Shannon diversity index, which accounts for both evenness and richness of a community. (**C**) Heatmap representing the relative abundances of the fifteen most abundantly present bacterialgenera. E: enzymes; BAC: intestinal bacteria (human faecal suspension). In A, mean and SD are shown of *n* = 3 independent determinations.

To assess the effect of PYC on the bacterial composition of retentate samples, Illumina HiSeq sequencing of PCR-amplified 16S rRNA gene amplicons was used (Figure 8). In the 4 h sample, the addition of PYC resulted in an increase for the genera *Collinsella* (d = +8% points), *Streptococcus* (+4%), and *Bifidobacterium* (+3%), and a decrease for *Eubacterium hallii* (−11%), *Anaerostipes* (−5%), and *Lachnospiraceae_UCG-004* (−5%). For the samples from time point 10 h, the PYC sample presented increased abundances for the genera *Enterococcus* (+5%) and *Streptococcus* (+4%), while *Blautia* (−9%), *Dorea* (−4%), and *Veillonella* (−4%) were decreased (Figure 8C). Moreover, no differences could be observed for *Collinsella*. *Eubacterium hallii* was present in the sample containing enzymes and bacteria only. Consistent changes at both time points were thus observed for *Streptococcus*, *Sutterella*, *Bacteroides*, and *Bifidobacterium,* which increased in the PYC sample, while relative abundances of *Dorea*, *Lachnospiraceae_UCG-004*, *Anaerostipes*, and *Eubacterium hallii* were decreased in the sample without PYC (Figure 8C).

Furthermore, alpha diversity was calculated using the Shannon index and Faith Phylogenetic diversity, which yielded results that were comparable between the different samples (Figure 8B). This suggests that the diversity of the community is stable, despite the changes in the bacterial composition. To determine whether the absolute number of bacteria was different due to PYC, real-time qPCR was performed. No differences in the absolute concentration of the bacteria between samples were observed (Figure 8A).

As expected, fingerprint chromatographic analysis of PYC GIDM dialysates (Figure S3) revealed a different composition of the metabolized sample compared to the PYC reference, as well as the control without PYC, due to microbial metabolism, though medium interference should also be taken into account. The exact composition of dialysates could not be revealed by HPLC analysis, due to the presence of various unknown compounds in PYC [4].

## 4. Discussion

TLRs on macrophages are involved in the inflammatory responses through the recognition of different structures of damage-associated molecular patterns (DAMPs) and pathogen-associated molecular patterns (PAMPs). Till now, 13 TLRs (TLR1-TLR13) have been described to be involved in various phases of the inflammatory responses, including release of proinflammatory cytokines/chemokines and antimicrobial peptides [35,36,37,38]. These signals attract immune cells, including macrophages, natural killer cells, and mast cells, which, as a consequence, may release reactive oxygen species (ROS) and reactive nitrogen species (RNS) [39]. ROS production not only directly causes cell injury and initiates associated degenerative processes, but can also act as signals for other processes, such as proinflammatory pathways involving NF-κB activation. The antioxidant properties of PYC have been elucidated in a variety of in vitro and in vivo studies [5,6,8,24,40,41,42,43,44,45]. In addition to its radical scavenging activity, inhibition of NF-κB-dependent gene expression, and inhibition of production and activity of various proinflammatory mediators and adhesion molecules was observed after the incubation of different cell types with PYC extract [13,17,19,21,26,46]. Despite many studies showing that PYC attenuates inflammation via suppressing oxidative stress, the mechanism of action remains largely unknown. The study of Luo et al. [25] showed that PYC attenuated LPS-induced lipid droplet formation through the TLR4 and NF-κB pathway. Additionally, other studies suggested the involvement of PYC in suppression of the TLR4-related proinflammatory cascade [26,46]. In our model system with TLR4 transfected HEK 293 cells, we did not see any inhibitory (antagonistic) action of PYC against LPS-induced TLR4 activation, which is in contradiction with the data presented in literature. The differences between our results and the outcomes of others [25,47], who showed that PYC reduces the TLR4 signaling, are most likely caused by mechanistic differences in the cell lines used in the different studies. Liu and colleagues used mouse macrophages, while we used HEK 293 cells transfected with human TLR4 [48]. There are a few possible mechanisms of blocking of the TLR signaling pathway, including (a) extracellular conformational blockers of the binding of selective ligands to the receptor, resulting in impaired signal transduction, and (b) intracellular small molecule inhibitors that block intracellular TLR signal transduction [49]. It has been reported that PYC blocks the TLR-JNK pathway, but the exact mechanisms of action, as well as the compounds responsible, have not been elucidated in detail. Therefore, it is possible that some of the small size PYC compounds are able to cross cell membranes and act on specific intracellular adapter proteins or compartments along the TLR signaling pathways, which has been described before in the literature as the mechanism of action of certain drugs. Such a mechanism includes the inhibition of MAPK signaling and phospholipase A2, anti-proliferative effects, and reduction of matrix metalloproteinase-9 (MMP-9) activity or binding to the intracellular TIR domain of TLR4, which all lead to diminished LPS-induced TLR4 signaling and inflammation [49,50]. Taking these possibilities into account, the HEK cells may not express the appropriate transporter(s) for certain PYC compounds, which could explain the differences in the observed results.

We found that PYC and CAT do not function as an agonist or antagonist for TLR4 on their own in the HEK 293 model. However, when provided in the presence of co-stimulators like LPS, PYC works as an agonist. We showed that PYC and LPS form complexes that show enhanced binding and/or activation of TLR4. It is likely that LPS and PYC will form complexes through hydrophobic interactions, as both compounds have large hydrophobic portions [51]. Previous studies confirm the potential of PYC to interact with proteins (xanthine oxidase) through hydrophobic bonding [52]. Although it is not known which compound(s) in the PYC preparation form complexes with LPS, our data indicate that it is not its main monomer CAT.

In the present study, we elucidated the involvement of TLR receptors other than TLR4 (TLR1/2, TLR2/6, and TLR5) in the immunomodulatory effects of PYC. Moreover, for the first time, the bioactivity of non-metabolized PYC was compared with microbially-metabolized PYC. We showed that non-metabolized PYC acts as an agonist of TLR1/2 and TLR2/6 and as a partial agonist of TLR5, though only in concentrations higher than 25 µg/mL. The agonistic effect of non-metabolized PYC against TLR1/2 and TLR2/6 may be responsible for the immunomodulatory effects when administrated orally. Therefore, in the presented work, we aimed to answer the question relevant for the in vivo situation, namely, what happens to the immunomodulatory properties of PYC after microbial metabolism.

Co-stimulation of HEK 293 cells expressing TLR1/2 and TLR2/6 with agonists of these receptors (PAM3 and PAM2) in the presence of PYC did not result in any reduction of receptor activation, suggesting no antagonistic action of PYC against these receptors. However, PYC dose-dependently inhibited the activation of TLR5 when HEK 293 cells were co-stimulated with flagellin. This finding suggests that PYC may be a partial agonist of TLR5 in the presence of which its full agonist (flagellin) is unable to bind to the receptor, leading to full activation. Without the presence of the full agonist, PYC acts as a ligand that binds to the TLR5 receptor binding site to induce some conformational change without leading to full activation of the receptor (Figure 3C). This phenomenon was already described for underacylated and underphosphorylated derivatives of lipid A that have partial TLR4 agonist properties, resulting in weak agonistic properties but also antagonistic properties in the presence of a full agonist [53,54].

The investigation of immunomodulatory properties of metabolized PYC concerned the analysis of (a) the retentate fraction and (b) the dialysate fraction. The retentate fraction of metabolized PYC showed a significantly lower level of activation of TLR1/2 and TLR2/6 compared to control samples with enzymes and bacteria only (Figure 5A–C). These data suggests that gastrointestinal metabolites of PYC (retentate fraction) show antagonistic or partial agonistic effects on TLR1/2 and TLR2/6. TLR1/2 and TLR2/6 signaling was impaired by PYC metabolites. Therefore, in the presence of PYC metabolites, the full agonist (bacteria and their fragments present in the retentate) was unable to bind to the receptor, leading to full activation observed in the control samples without PYC. This effect was not caused by the differences in bacterial numbers in the samples (Figure 8A), but might be partially explained by differences in microbiota composition caused by PYC (Figure 8C).

The dialysate part of the PYC metabolites at time point 4 h was able to activate TLR1/2 and TLR2/6, but this effect was not observed for any further time points of metabolism (Figure 5B,D). However, the activation was small (less than 5-fold), albeit significant, as compared to more than 40-fold increased activation with retentate fractions. When THP-1 macrophages were exposed to dialyzed PYC metabolites, an enhanced expression of TLR1 and TLR5 was observed for time point 4 h compared to cells exposed to the control samples with enzymes and bacteria only. These findings suggest that metabolites of PYC can activate TLR-dependent signal transduction pathways, but this activation is limited, as reflected in the almost complete absence of proinflammatory cytokines levels. However, enhanced IL-10 secretion was observed by THP-1 macrophages incubated with metabolized PYC (dialysate fraction). IL-10 is often secreted as a feedback inhibitor during inflammation initiated by LPS or tissue damage [55]. However, in our study, the levels of proinflammatory cytokines were not altered Figure 6. An increased level of IL-10 secreted by THP-1 macrophages may be explained by the activation of TLRs, as it was already shown for intestinal epithelial cells [56]. Nevertheless, additional studies are needed to confirm this hypothesis. An increased release of IL-10 by THP-1 macrophages upon stimulation with the dialysate of PYC metabolites confirms the anti-inflammatory properties of PYC reported by other studies [7,8,11,12,13,15,27,57,58,59,60] and suggests that PYC may help in balancing intestinal homeostasis, especially during chronic inflammatory conditions. Because PYC is a complex mixture of polyphenols, it is difficult to speculate about the specific compounds/metabolites, which may exert the described effects. One of the known metabolites of CAT called M1 (δ-(3,4-Dihy-droxy-phenyl)-γ-valerolactone) was already shown to be taken up by macrophages via facilitated transport, where it accumulates and undergoes further intracellular metabolism to levels that could be bioactive [4,23,61]. Our results showed that CAT acts as antagonist of TLR4 and TLR5, but only at the high concentration (250 µg/mL). In addition, PYC was shown to display stronger biological activity as a mixture than when separated into its individual components, indicating that the components interact synergistically [62].

Another explanation for the observed inhibition of TLRs signaling by PYC may be the influence of PYC and its metabolites on microbiota composition. Although this result needs to be interpreted with caution because a strong effect of time of the digestion on microbiota composition was also observed, the sample containing PYC showed differences in microbial composition when compared to the control sample for both analyzed time points. It was observed that PYC modulates the microbial composition by increasing the presence of Bifidobacterium, Streptococcus, Sutterella, Bacteroides, and Enterococcus after 10 h of digestion. Therefore, the different diversity of PAMPs may also influence the levels of activation of the different TLRs by the metabolized samples. There are currently no data on the specific effect of PYC on microbiota composition, but it was shown that dietary polyphenols have an effect on gut microbiota [63,64,65,66], as well as on the expression of TLRs on immune cells [64]. Here, we showed that metabolites of PYC are able to modify the microbial composition and inhibit the TLR signaling (TLR1/2, TLR2/6), although downstream signaling mechanisms of these activities need to be further investigated.

## 5. Conclusions

In conclusion, our study showed that non-metabolized PYC acts as an agonist of TLR1/2 and TLR2/6 receptors, as well as a partial agonist of TLR5. Microbial digestion of PYC reveals the biological activity of PYC metabolites as potential inhibitors of TLR1/2 and TLR2/6 signaling (retentate fraction) and inducers of IL-10 secretion from THP-1 macrophages (dialysate fraction). Moreover, PYC was shown to influence the intestinal microbiota composition, though a more detailed study, including larger numbers of feces donors, is required in order to get more insight and understanding into the microbiota composition promoted by the presence of PYC metabolites. Based on the presented data, microbially-metabolized PYC can show local gut (retentate), but also systemic (dialysate), immunomodulatory effects via: (1) inhibition of Toll-like receptor (TLR) signaling by acting as an antagonist or a partial agonist; (2) alteration of microbiota composition, by directly or indirectly (via metabolites) affecting specific microbial growth; (3) affecting signal transduction, by interfering with transcription factors involved in inflammatory and immune response activation (upregulation of surface markers on immune cells). The molecular mechanisms underlying these immunomodulatory activities are not completely characterized and need to be elucidated in further studies. Our data represent a pioneer study on the potential role of PYC in promoting health condition due to its immunomodulatory activity, next to its well-documented antioxidant and free radical scavenging properties, when used as a dietary supplement.

## Figures and Tables

**Table 1:** Solubility of catechin, caffeic acid, taxifolin, and ferulic acid at different concentrations of PYC (1, 4 and 50 mg/mL) in PBS (Phosphate Buffered Saline) referred to solubility in methanol (=100%) determined by high-performance liquid chromatography (HPLC).

	% Catechin			% Caf Acid			% Taxifolin			% Fer Acid		
PYC conc (mg/mL)	1	4	50	1	4	50	1	4	50	1	4	50
HPLC after solubilisation	93.3	97.6	93.6	89.7	96.3	93.3	88.8	93.9	84.3	96.6	99.5	86.2
HPLC after solubilisation and 15 min sonification	91.6	100.0	87.9	88.1	99.4	95.1	86.7	96.4	84.0	96.6	101.5	84.0
HPLC after solubilisation and filtration	96.1	98.0	88.8	91.3	96.7	92.2	92.1	95.0	80.4	102.5	99.8	82.7

## Supplementary Materials

The following are available online at http://www.mdpi.com/2072-6643/11/2/214/s1, Figure S1. Ethanol solution of PYC impairs valid TLR modulation of TLR1/2 and TLR5 receptors when compared to PBS solution of PYC. An effect of solvent on the stimulation of TLR2/1 and TLR5 by PYC solution was compared using the transfected HEK 293 cell line. HEK293 cells expressing the receptors (A-B) TLR1/2 or (C-D) TLR5 were incubated 24 h with different concentrations of PYC and CAT or their known ligands as positive controls (LTA and flagellin, resp.). All corresponding positive ligands induced TLR activation, confirming functionality of the assays, Figure S2: Non-metabolized PYC dose-dependently induces proinflammatory cytokine secretion from THP-1 macrophages. THP-1 macrophages were incubated for 24 h with increasing concentration of PYC or CAT, and the concentration of IL-8, TNF-α, IL-1β, and IL-6 in supernatant was determined with flow cytometry (A–E), Figure S3: Fingerprint chromatogram of GIDM dialysate samples.
