# Supplementary material for: Toll-Like Receptor-Dependent Immunomodulatory Activity of Pycnogenol®
Source: Nutrients. 2019 Jan 22;11(2):214. doi: 10.3390/nu11020214 (PMC6412808; doi:10.3390/nu11020214)
Supplement: Supplementary file 1 [file nutrients-11-00214-s001.pdf]

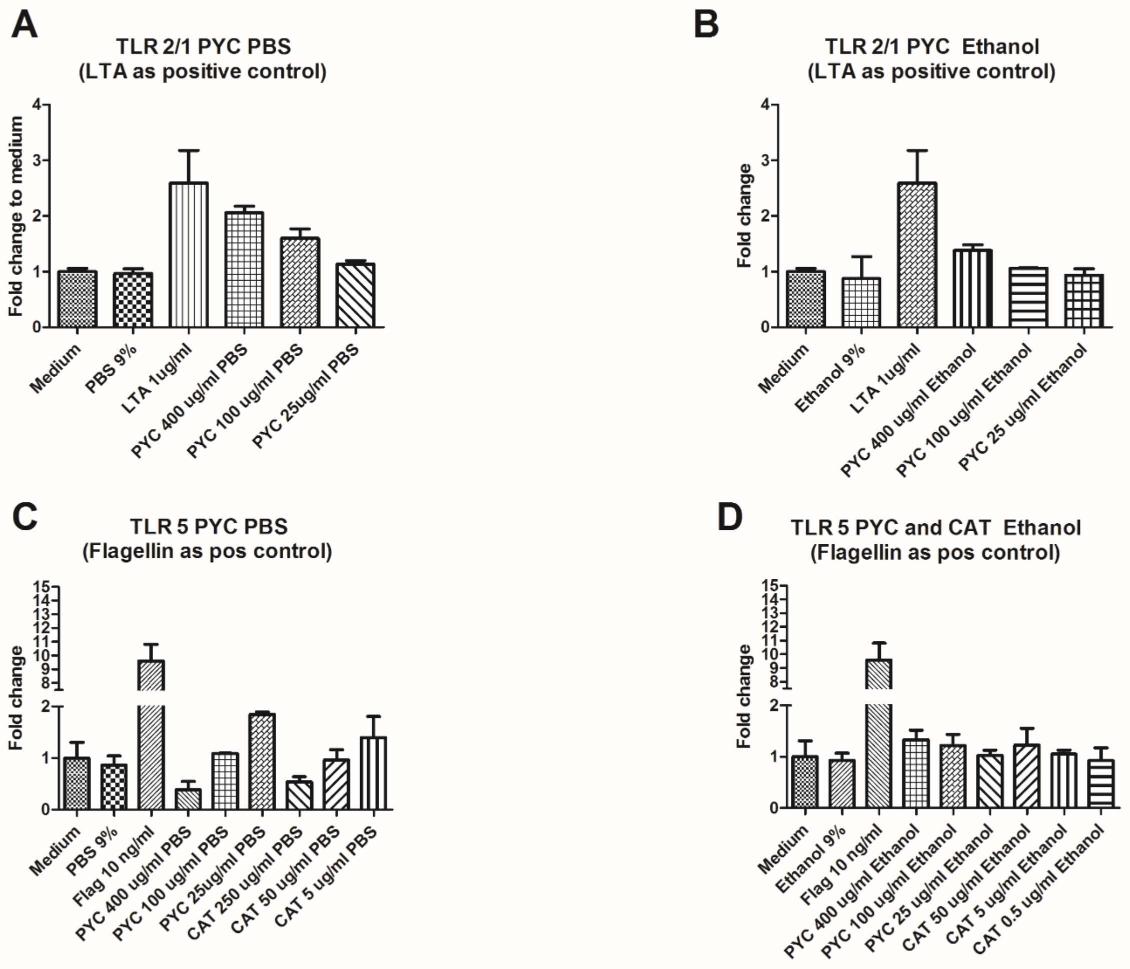

Figure S1. Ethanol solution of PYC impairs valid TLR modulation of TLR1/2 and TLR5 receptors when compared to PBS solution of PYC. An effect of solvent on the stimulation of TLR2/1 and TLR5 by PYC solution was compared using the transfected HEK 293 cell line. HEK293 cells expressing the receptors (**A,B**) TLR1/2 or (**C,D**) TLR5 were incubated 24h with different concentrations of PYC and CAT or their known ligands as positive controls (LTA and flagellin, resp.). All corresponding positive ligands induced TLR activation, confirming functionality of the assays. Results are expressed as fold change of fluorescence intensity to unstimulated cells cultured in medium as control (*n* = 3 as technical replicates). All data are expressed as the mean ± SD.

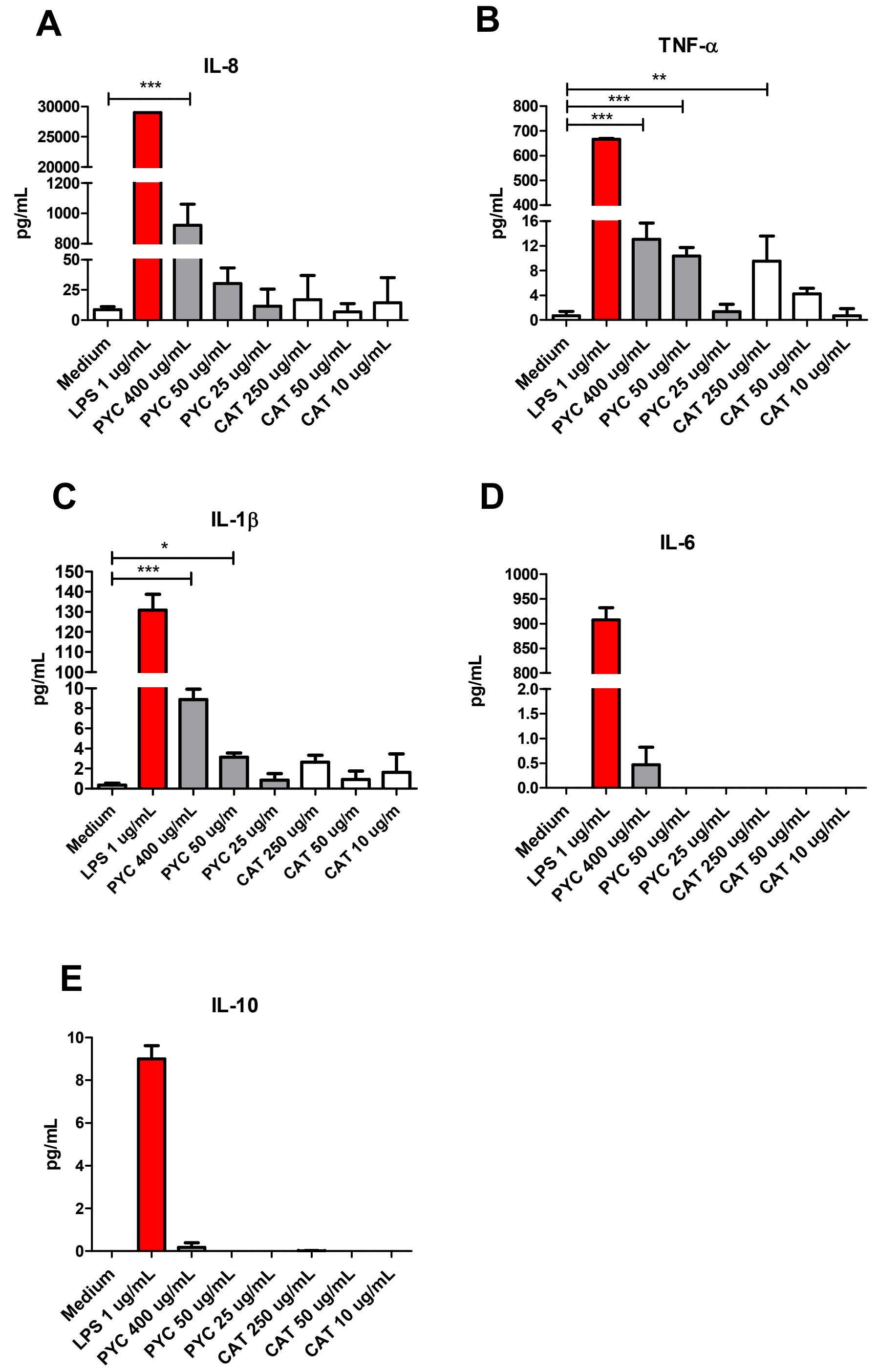

Figure S2. Non-metabolized PYC dose-dependently induces pro-inflammatory cytokine secretion from THP-1 macrophages. THP-1 macrophages were incubated 24h with increasing concentration of PYC or CAT, and the concentration of IL-8, TNF-α, IL-1β and IL-6 in supernatant was determined with flow cytometry (**A**–**E**). All data (n = 4 technical replicates) are expressed as the mean ± SD. P-values < 0.05 are considered statistically significant as analyzed with one-way ANOVA with Tukey post hoc comparison test (GraphPad Prism). Significant differences are indicated by asterisks: * $p < 0.05$; ** $p < 0.01$; *** $P < 0.001$.PYC dose-dependently enhances TLR4 activation when co-stimulated with LPS.

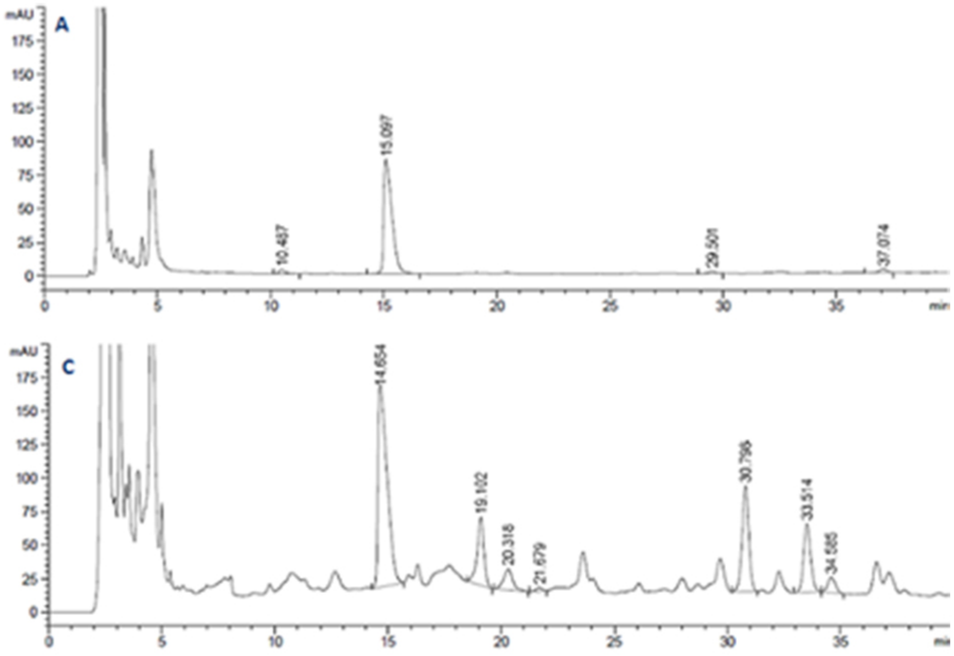

Figure S3. Fingerprint chromatogram of GIDM dialysate samples: (**A**) blank with enzymes and bacteria, (**C**) sample with PYC, enzymes and bacteria.
